# Pembrolizumab as first-line treatment for metastatic uveal melanoma

**DOI:** 10.1007/s00262-019-02352-6

**Published:** 2019-06-07

**Authors:** Ernesto Rossi, Monica Maria Pagliara, Daniela Orteschi, Tommaso Dosa, Maria Grazia Sammarco, Carmela Grazia Caputo, Gianluigi Petrone, Guido Rindi, Marcella Zollino, Maria Antonietta Blasi, Alessandra Cassano, Emilio Bria, Giampaolo Tortora, Giovanni Schinzari

**Affiliations:** 1 Department of Medical Oncology, Fondazione Policlinico Universitario “A. Gemelli” IRCCS, Università Cattolica del Sacro Cuore, L.go F. Vito 1, 00168 Rome, Italy; 2 Department of Ophthalmology, Fondazione Policlinico Universitario “A. Gemelli” IRCCS, Università Cattolica del Sacro Cuore, L.go F. Vito 1, 00168 Rome, Italy; 3 Department of Pathology, Fondazione Policlinico Universitario “A. Gemelli” IRCCS, Università Cattolica del Sacro Cuore, L.go F. Vito 1, 00168 Rome, Italy; 4 Institute of Genomic Medicine, Fondazione Policlinico Universitario “A. Gemelli” IRCCS, Università Cattolica del Sacro Cuore, L.go F. Vito 1, 00168 Rome, Italy

**Keywords:** Uveal melanoma, Immunotherapy, Pembrolizumab, First line, Liver metastases

## Abstract

**Background:**

No standard treatment has been defined for metastatic uveal melanoma (mUM). Although clinical trials testing Nivolumab/Pembrolizumab for cutaneous melanoma did not include mUM, anti PD-1 agents are commonly used for this disease.

**Patients and methods:**

In this prospective observational cohort single arm study, we investigated efficacy and safety of Pembrolizumab as first-line therapy for mUM. The efficacy was evaluated in terms of progression-free survival (PFS), response rate and overall survival (OS). Toxicity was also assessed.

**Results:**

Seventeen patients were enrolled. A median of 8 cycles were administered (range 2–28). Two patients achieved partial response (11.7%), 6 a disease stabilization (35.3%), whereas 9 (53%) had a progression. No complete response was observed. PFS of the overall population was 3.8 months. PFS was 9.7 months for patients with an interval higher than 5 years from diagnosis of primary tumor to metastatic disease and 2.6 months for patients with an interval lower than 5 years [*p* = 0.039, HR 0.2865 (95% CI 0.0869–0.9443)]. Median OS was not reached. The two responding patients were still on treatment with Pembrolizumab at the time of data analysis. Survival was 12.8 months for patients with clinical benefit, while OS for progressive patients was 3.1 months. PD-L1 expression and genomic abnormalities predictive of relapse after diagnosis of primary tumor were not associated with PFS. Toxicity was mild, without grade 3–4 side effects.

**Conclusions:**

The efficacy of Pembrolizumab does not seem particularly different when compared to other agents for mUM, but responding patients had a remarkable disease control.

## Introduction

Uveal melanoma is a rare tumor, but the most common malignancy of the eye [1]. Metastatic spread is frequent [2] despite radical treatment of the primary tumor. The liver is the first metastatic site as hepatic involvement represents about 90% of the metastatic disease [3, 4]. Liver metastases are usually multifocal, limiting the possibility of a loco-regional procedure [5]. The prognosis of metastatic disease is dismal for the majority of patients and no standard treatments have been established. Several approaches for metastatic uveal melanoma have been proposed, although they often followed clinical trials on cutaneous melanoma. Among the systemic therapies, chemotherapy was used with poor results, considering the median overall survival (OS) lower than 15 months [6–8]. Target agents were tested without benefits [9]. After the introduction for the treatment of cutaneous melanoma, immune checkpoint inhibitors have also become a common therapy for uveal melanoma, despite controlled trials with immunotherapy did not include ocular melanoma. Ipilimumab is associated with a slight activity both in pre-treated and in naïve patients with metastatic uveal melanoma [10–12]. In pre-treated patients, Karydis et al. [13] reported a partial response rate of 8% and disease stabilization of 24% with Pembrolizumab, while the median progression-free survival (PFS) was 3 months. Similar results were described by Algazi et al. [14], who employed Pembrolizumab, Nivolumab or atezolizumab in 48 pre-treated patients and 8 naïve patients. There are currently no available prospective clinical data with immune checkpoint inhibitors in a population of only naïve patients.

We have hereinafter described the preliminary results of a prospective observational cohort single-arm study of Pembrolizumab as first-line treatment in patients with metastatic uveal melanoma.

## Patients and methods

### Patients

This prospective study included patients with histologically proven advanced uveal melanoma. An Eastern Cooperative Oncology Group (ECOG) performance status of 0–2, adequate bone marrow, liver and renal functions were required. No prior systemic anticancer treatments were allowed. Inclusion and exclusion criteria are outlined in Table 1.

**Table 1:** Inclusion and exclusion criteria

Inclusion criteria
Histologically confirmed advanced uveal melanoma
At least one measurable metastases as per RECIST 1.1
Age ≥ 18 years
ECOG performance status ≤ 2
Hemoglobin ≥ 12.0 g/dl; platelets ≥ 100 × 10^9^/l; WBC ≥ 3.0 × 10^9^/l
AST and ALT ≤ 2.5 ULN
Total bilirubin ≤ 2.0 mg/dl
Exclusion criteria
Prior systemic anticancer treatment
History of other neoplasm
Instable heart failure
Serious respiratory failure
History of rheumatic disease
Chronic use of systemic corticosteroids

*RECIST* response evaluation criteria in solid tumors, *ECOG* Eastern Cooperative Oncology Group, *AST* aspartate aminotransferase, *ALT* alanine aminotransferase

Pembrolizumab was administered intravenously at the dose of 2 mg/kg every 3 weeks, until disease progression, unacceptable toxicity or consent withdrawal. Toxicity was reported according to Common Terminology Criteria for Adverse Events (CTCAE vs 4.0). Baseline tumor assessment with brain–chest–abdominal computed tomography (CT) scan or brain and abdominal magnetic resonance imaging (MRI) was required within 3 weeks before the first Pembrolizumab administration. Subsequent tumor assessment was scheduled every 9 weeks until disease progression or discontinuation for any reason, with the possibility of anticipating the radiological evaluation in case of signs and symptoms of tumor progression.

The primary endpoint of the study was PFS. An interim analysis was planned after the observation of at least 50% cases of progression. Secondary endpoints were response rate, clinical benefit, OS and tolerability. Response was assessed according to RECIST criteria 1.1. The response rate included complete and partial responses. Clinical benefit was defined as the percentage of patients with a complete or partial response or disease stabilization. PFS was calculated from the first day of treatment to progression or death for any reason. Survival was defined as the interval from the first day of treatment to death for any cause. PFS and OS were calculated with the Kaplan–Meier method. The log-rank test was employed to analyze the differences between patient subgroups.

### Immunohistochemical evaluation

Formalin-fixed paraffin-embedded (FFPE) tissue blocks were retrieved from the archives of the Department of Pathology, Fondazione Policlinico Universitario Agostino Gemelli IRCCS, Rome. PD-1 and PD-L1 expression was evaluated using the primary Abs PD-1 (monoclonal mouse, NAT105, Ventana, prediluted) and PD-L1 (Kit DAKO, Monoclonal mouse, clone 22C3 PharmDx, prediluted). The slides were independently evaluated and subsequently analyzed by two experienced pathologists. The expression of PD-L1 and PD-1 was tested on primary tumor or liver metastasis or both, when available.

PD-L1 positivity was defined by a threshold of 5% of tumor cell expression. Positive cells had a strong cytoplasmic expression with membrane-accentuating or single membrane pattern [15]. Inner control was determined by cytoplasmic positivity for retinal pigmented epithelium cells as previously described [16].

### Genetic analysis

DNA from uveal melanoma samples, fresh frozen or formalin fixed/paraffin embedded tissues, was extracted using DNeasy Blood & Tissue Kit (QIAGEN, Hilden, Germany). Oligonucleotide aCGH was performed using the Agilent Human Genome CGH microarray 8X60K (Agilent Technologies Santa Clara, CA, USA), with an average resolution of 75 kb, following the manufacturer’s instructions.

The arrays were analyzed with the SureScan Microarray Scanner, a graphical overview of the results was obtained using CytoGenomics V2.7.22.0 software (Agilent Technologies Santa Clara, CA, USA).

## Results

From January 2016 to April 2018 the study enrolled 17 patients, whose characteristics are summarized in Table 2. The median age was 64.7 years (ranging between 29 and 83). Nine subjects were males and 8 females. All the patients had hepatic metastases. None of them had resectable liver metastases. Seven patients had both hepatic and extra-hepatic disease. Ocular enucleation was previously performed for the treatment of primary tumor in 12 patients, while Ruthenium 106 brachytherapy was carried out in 5 patients. Four patients received a previous local treatment for liver metastases (metastasectomy or local ablation) at least 6 months before being recruited in the study. These patients progressed following local treatment and had a measurable disease when enrolled.

**Table 2:** Patients’ characteristics

Median age (range)	63.8 years (29–83)
	*N*. of patients
M/F	9/8 pt
Enucleation for primary tumor	12
Interval from diagnosis of primary to metastases	
> 5 years	7
< 5 years	10
Previous local treatment for liver metastases	4
Site of metastases	
Liver	17
Lung	6
Bone	2
Other	2
Hepatic and extra-hepatic metastases	7
BRAF mutation	0

A median of 8 cycles per patient was administered (range 2–28).

**Fig. 1:**
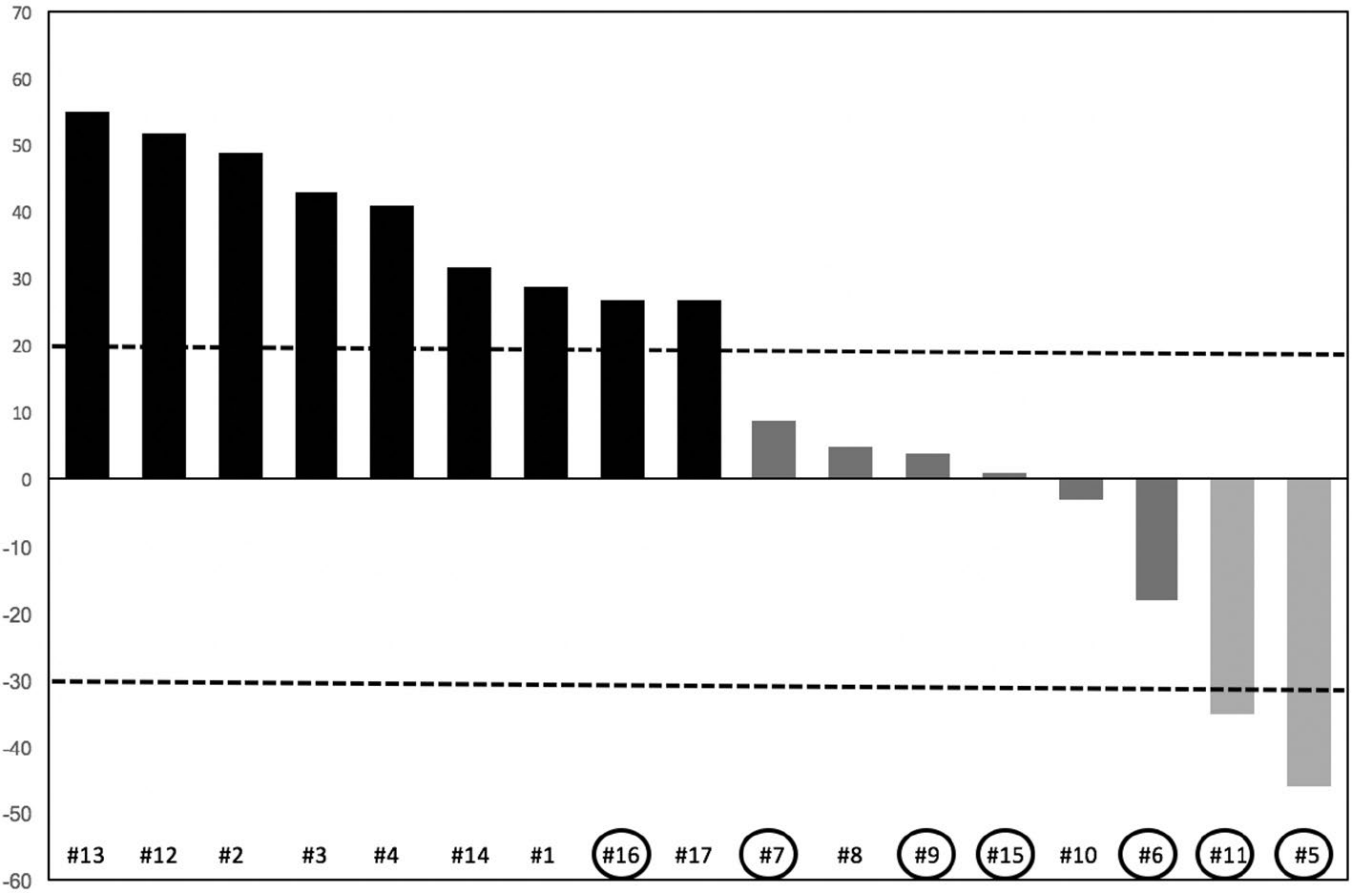
Waterfall chart of response. Black column: progression. Dark gray column: stable disease. Light gray column: partial response. Circles identify patients with an interval longer than 5 years from diagnosis of primary tumor to metastases

Two patients achieved a partial response (11.7%), 6 patients obtained disease stabilization (35.3%), while 9 (53%) patients showed disease progression at first tumor assessment (Fig. 1). The two responding patients without progression after 19.4 and 28.9 months, respectively, were still on treatment at the time of data analysis. Eight patients (47%) achieved clinical benefit. No complete response was reported.

**Fig. 2:**
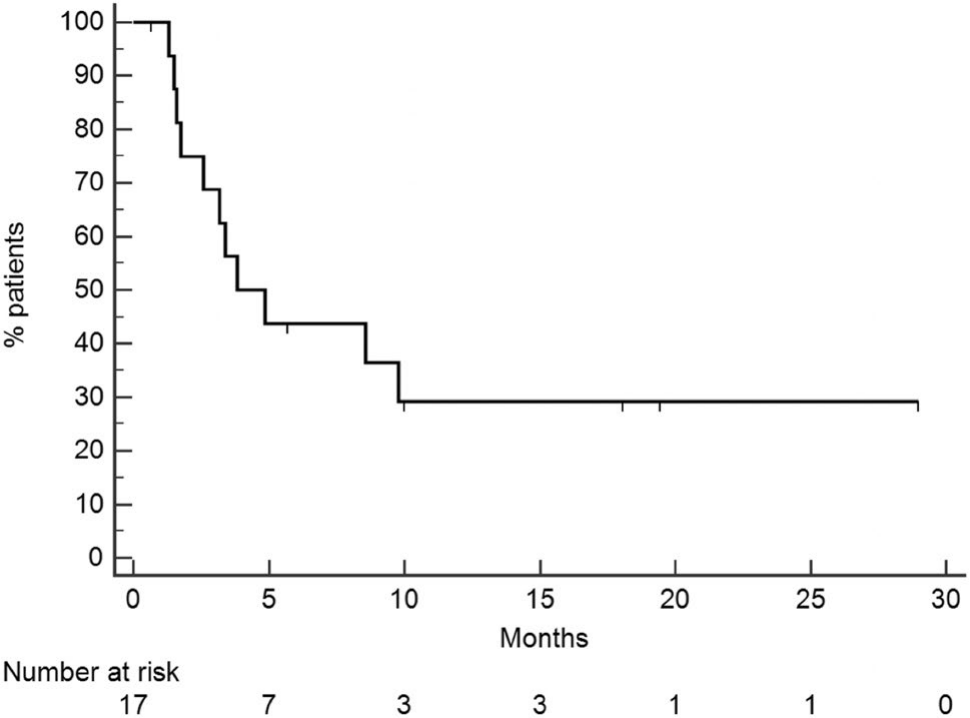
PFS of the entire population

**Fig. 3:**
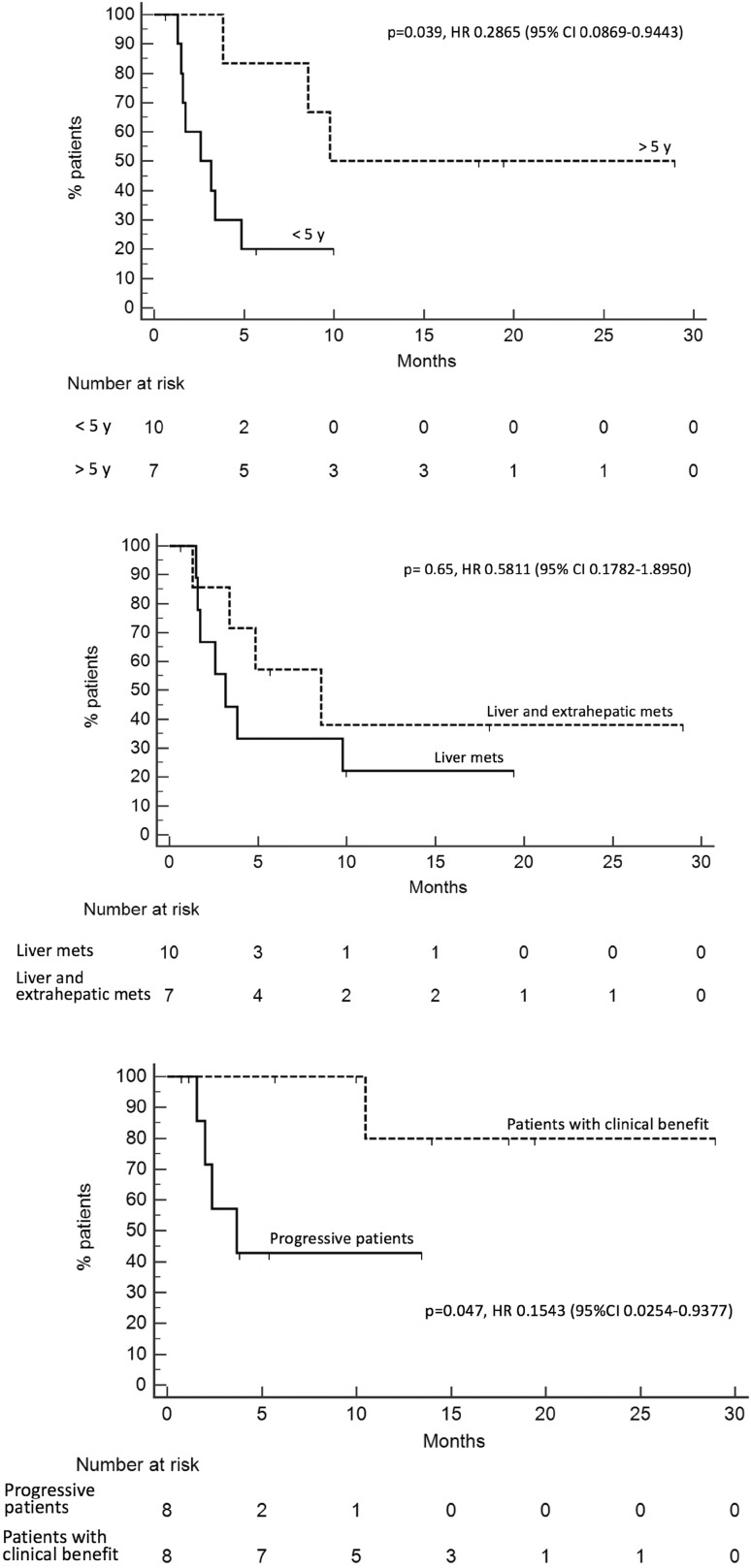
Stratification of time-dependent clinical endpoints. **a** PFS of patients with an interval longer than 5 years from diagnosis of primary tumor to metastatic disease (dashed line) vs patients with an interval lower than 5 years (solid line)—*p* = 0.039, HR 0.2865 (95% CI 0.0869–0.9443). **b** PFS of patients with only liver metastases (solid line) vs PFS for patients with liver and extrahepatic disease (dashed line)—*p* = 0.65, HR 0.5811 (95% CI 0.1782–1.8950). **c** Survival of patients with clinical benefit (dashed line) vs survival of progressive patients (solid line)—*p* = 0.047, HR 0.1543 (95% CI 0.0254–0.9377)

PFS of the whole population (Fig. 2) was 3.8 months (95% CI 2.9–9.7). Patients with an interval from diagnosis of primary tumor to metastatic disease longer than 5 years had a PFS of 9.7 months, whereas for patients with an interval lower than 5 years PFS was 2.6 months [*p* = 0.039, HR 0.2865 (95% CI 0.0869–0.9443) Fig. 3a]. PFS was 3.1 months for patients with only liver metastases, while, for patients with liver and extrahepatic metastases, it was 8.4 months [*p* = 0.65, HR 0.5811 (95% CI 0.1782–1.8950) Fig. 3b]. One of the two responding patients had only hepatic disease, while the other patients had both liver and extra-hepatic metastases. For the first patient the response was observed in liver metastases and in all the metastatic sites (liver, pancreas, pleura and lung) for the other patients. Ocular enucleation, previous local treatment and LDH did not influence PFS.

At the time of data cut-off for interim analysis, five deaths were reported. Therefore, median OS of the whole population was not reached. Survival for the patients with clinical benefit was 12.8 months and 3.1 months for patients with disease progression [*p* = 0.047, HR 0.1543 (95% CI 0.0254–0.9377) Fig. 3c].

No grade 3–4 adverse events were observed. Grade 2 hypophysitis was observed in one patient and grade 1 hypothyroidism was reported in 2 patients. No diarrhea, rash or other cutaneous adverse events occurred.

### PD-1 and PD-L1

We evaluated PD-1 and PD-L1 expression in 14 primary tumors and 10 metastases. PD-1 was negative in 100% of primary tumors and 90% of metastases. PD-L1 was negative in 92.9% of primary tumors and 90% of liver metastases. One of the responding patients did not express PD-L1 and PD-1 either in primary uveal melanoma or liver metastases. Liver metastases of the other responding patient were PD-1 and PD-L1 negative, while his primary tumor sample was not available.

### Genetic analysis

Genomic abnormalities are reported in Table 3. Samples for genetic analysis were available for 12 patients and no significant correlation was found between genomic alterations and PFS. Among the responding patients, chromosome 3 monosomy was found in association with 8q duplication in one of the patients.

**Table 3:** Genetic abnormalities

Pts	Monosomy 3	8q duplication^a^	Gross rearrangements: monosomy, trisomy, WCAD and/or PCAD	Other CNVs	TOT VAR
#2	Y	Y	5 (1q+,8p−,16p+,16q−,18q−)	2	9
#3	Y	Y	3 (1p−,16q−,21q−)	20	25
#4	Y	Y	2 (8p−,−19)	28	32
#5	Y	Y	2 (1p−,4q−)		4
#6	Y	Y	1 (17p−)	1	4
#7	Y		6 (1p−,+7,−11,−14,−15,−21)		7
#8	Y	Y	4 (4p+,7p+,8p−,16q−)	8	14
#9			5 (1p−,6p+,6q−,13q+,16q−)		5
#10	Y	Y	2 (6q−,8p−)	2	6
#11		Y	8 (1q+,4q+,6p+,6q−8p−,9p−,9q+,12p−)	4	13
#14		Y	5 (1p−,6p+,6q−13q+,16q−)	8	14
#17	Y	Y	4 (4p+,8p−,16q−,+19)	15	21

*CNVs* chromosome number variants, *TOT VAR* total variants, *WCAD* whole chromosome arm deletion/duplication, *PCAD* partial chromosome arm deletion/duplication

^a^Entire arm, partial or complete chromosome 8 trisomy

## Discussion

Metastatic uveal melanoma is a poor-prognosis disease. To date, remarkable advantages of a systemic therapy have not been reported and a standard treatment has not been established. Although uveal melanoma was ruled out in controlled clinical trials, this neoplasm is commonly treated as a cutaneous melanoma, despite the different clinical and biological features. BRAF is often wild type, thus also excluding the possibility of a treatment with BRAF/MEK inhibitors.

Immunotherapy is one of the options for this disease, particularly chosen for the favorable toxicity profile. The available studies on anti-PD-1 agents in mUM are retrospective and included pre-treated patients [13, 14, 17], while there is a lack of prospective data on naïve patients only. We have evaluated Pembrolizumab, an anti-PD-1 monoclonal antibody, as first-line treatment for advanced uveal melanoma.

In the present study, the response rate was 11.7%. More than half of the patients experienced a progression at first tumor assessment. The objective response rate was lower than in cutaneous melanoma treated with pembrolizumab (32.9%), despite the different schedule used [18]. In our previous study on the efficacy and safety of a triple-agent chemotherapy, we found a 20% response rate, a 68% clinical benefit and a survival advantage for patients achieving tumor control [19]. The results in the chemotherapy study could be justified by the selected population suitable for a cisplatin-based combination chemotherapy.

In our population treated with Pembrolizumab, two responding patients were still alive for at least 19 months, suggesting a possible survival advantage in case of tumor shrinkage.

The median PFS of the entire population in our study (3.8 months) was similar to the PFS reported by Karidis et al. [13] (91 days) and slightly longer than the PFS described by Algazy et al. [14] (2.6 months) in their retrospective analyses.

The patients with an interval from the diagnosis of primary tumor to metastases longer than 5 years had a significant prolonged PFS compared to patients with an interval lower than 5 years (9.8 months vs 3.7 months). In UM, relapse may occur more than 5 years after treatment of the primary tumor [20]. It was suggested that immune-surveillance through T cell activity is a mechanism which explains the late recurrence [20, 21]. Therefore, our aim was to verify the different efficacy of immunotherapy in patients relapsing within 5 years compared to patients with later recurrence. Based on our data, relapse after 5 years from the diagnosis of primary tumor could be considered a parameter to select patients who may benefit more from immunotherapy.

In our study, patients with extra-hepatic disease showed a longer PFS than patients with only liver metastases. Although not statically significant, this datum seems to confirm the results in pre-treated patients [3], suggesting a role of immunotherapy mainly in patients with extra-hepatic disease. The possibility of immune escape in patients with only liver metastases could justify this observation [22].

We confirmed the favorable toxicity profile of Pembrolizumab in metastatic uveal melanoma.

The small number of patients responding to Pembrolizumab showed a remarkable survival advantage. Therefore, the identification of predictive factors for response is crucial. In our study, basal LDH did not correlate with PFS, in contrast with the results reported by Heppt et al. [17]. As possible predictive factors, we also evaluated both PD-L1 expression and genomic abnormalities commonly involved in the risk of relapse after the diagnosis of primary UM.

In our series, we found a limited expression of PD-L1 both in primary tumor and in metastases (7.1% and 10%, respectively). Few reports are available on PD-L1 expression in UM. Zoroquiain et al. [15] reported a 40% PD-L1 expression on tumor cells of primary UM in patients with metastatic disease. On the other hand, similar to our data, Javed et al. [23] observed that only 5% of uveal melanoma expresses PD-L1 in the metastatic sites. The responders in our study did not express PD-L1 either in primary tumor or metastases, thus PD-L1 could not be considered a predictive factor for these patients.

Regarding the genetic results, different genomic alterations, such as chromosome 3 monosomy and gains of chromosome 8q, are predictive of relapse after primary treatment [24–28]. In this study, we analysed the association between these genomic alterations with PFS. The genomic alterations found in 12 metastatic patients did not allow to identify specific abnormalities correlated with PFS. This result seems to suggest that the genetic prognostic factors, commonly predictive of relapse after treatment of the primary tumor, are not useful in predicting the efficacy of Pembrolizumab for metastatic disease.

It has recently been reported that a defect of MBD4, a transcriptional factor of gene promoters, is associated with a hypermutated CpG > TpG pattern which generates multiple subclones of the primary UM with more heterogeneous metastases. A patient with this MBD4-related hypermutator phenotype showed a remarkable response to immune checkpoint inhibitors [29]. Indeed, this evidence implies that specific and selected subgroups of UM could benefit from immunotherapy.

Due to the rarity of the disease, the small sample size of the study limits the interpretation of the data. Results regarding survival and patients still on treatment could provide further information.

Pembrolizumab is a well-tolerated agent which allows to obtain an objective response in metastatic uveal melanoma only in a minority of patients. These patients could benefit from a long-term disease control. Further investigations on the biological characteristics of uveal melanoma are essential to select patients who could benefit more from immunotherapy.

## Abbreviations

LDH: Lactate dehydrogenase
mUM: Metastatic uveal melanoma
OS: Overall survival
PD-1: Programmed death 1
PD-L1: Programmed death ligand 1
PFS: Progression free survival
UM: Uveal melanoma
